# HucMSC‐Derived Exosomes Preserve Mafb‐Dependent Tubular Epithelial Identity and Suppress Dedifferentiation in Obstructive Nephropathy

**DOI:** 10.1002/pdi3.70059

**Published:** 2026-06-30

**Authors:** Zhuocheng Shi, Chenxi Jia, Meiling Chen, Yihang Yu, Deying Zhang, Guanghui Wei

**Affiliations:** ^1^ Department of Urology Children's Hospital of Chongqing Medical University, National Clinical Research Center for Child Health and Disorders, Ministry of Education Key Laboratory of Child Development and Disorders Chongqing China; ^2^ Chongqing Key Laboratory of Structural Birth Defect and Reconstruction Chongqing China; ^3^ Children Urogenital Development and Tissue Engineering of Chongqing Education Commission of China Chongqing China

## Abstract

Tubular epithelial dedifferentiation and loss of polarity are early pathological events in obstructive nephropathy and critically contribute to progressive renal fibrosis. Human umbilical cord mesenchymal stem cell–derived exosomes (HucMSC‐Exos) have shown therapeutic potential for kidney injury, yet the mechanisms by which they preserve epithelial identity remain unclear. Mafb, a transcription factor essential for renal epithelial differentiation, has been implicated in epithelial homeostasis, but its role in adult obstructive injury and its regulation by exosomes are unknown. Here, a unilateral ureteral obstruction (UUO) mouse model was used to evaluate early tubular injury and the effects of HucMSC‐Exos. UUO induced marked epithelial damage characterized by decreased E‐cadherin, increased vimentin and α‐smooth muscle actin (α‐SMA) expression, and disruption of epithelial polarity, whereas HucMSC‐Exo administration significantly ameliorated these alterations. Mafb expression was profoundly downregulated in UUO kidneys but was effectively restored following exosome treatment. In vitro, *Mafb* knockdown in Human renal proximal tubular epithelial (HK‐2) cells markedly aggravated TGF‐β1–induced dedifferentiation, confirming its protective role in epithelial integrity. Analysis of *Mafb* ^−/−^ embryonic kidneys demonstrated severe developmental defects and loss of polarity markers, highlighting the essential function of *Mafb* in renal epithelium. Importantly, HucMSC‐Exos rescued epithelial marker expression even in *Mafb*‐silenced cells, suggesting both *Mafb*‐dependent and independent mechanisms. These findings identify *Mafb* as a key regulator of tubular epithelial homeostasis and reveal that HucMSC‐Exos attenuate early renal injury partly through Mafb restoration.

## Graphical abstract

Unilateral ureteral obstruction (UUO) and genetic deletion of *Mafb* lead to tubular epithelial dedifferentiation, characterized by decreased E‐cadherin and increased α‐SMA and vimentin expression. Intravenous administration of human umbilical cord mesenchymal stem cell‐derived exosomes (HucMSC‐Exos) partially restores Mafb expression in UUO kidneys, preserves epithelial polarity, and attenuates renal fibrosis. These findings reveal Mafb as a key regulator of tubular epithelial integrity and identify HucMSC‐Exos as a potential therapeutic strategy for chronic kidney disease.

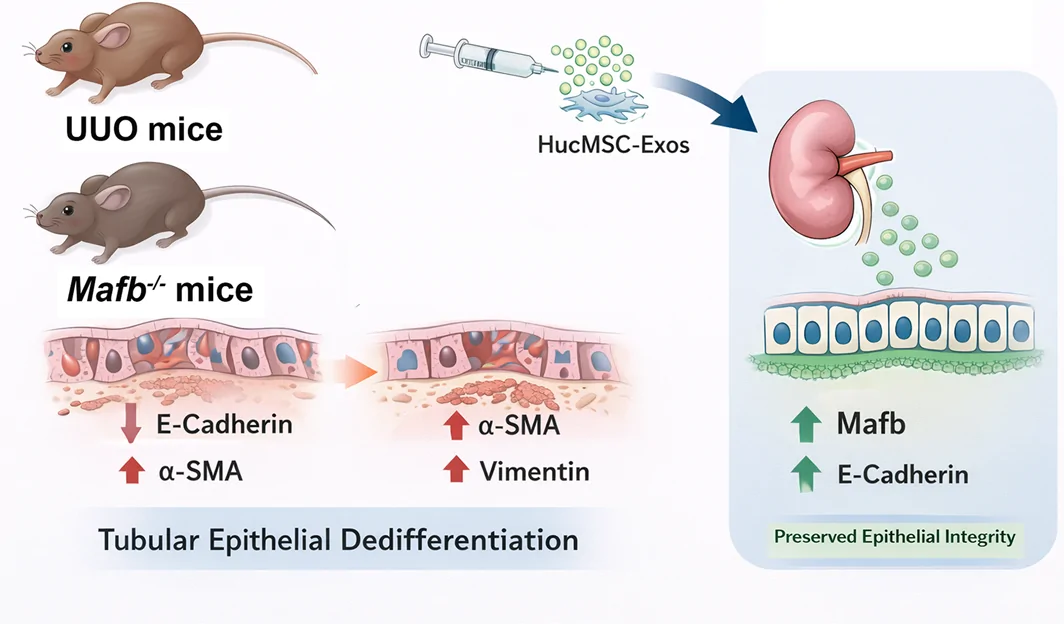

## 1 Introduction

Renal fibrosis is the final common pathway of chronic kidney disease (CKD) regardless of etiology and is characterized by progressive tubular epithelial injury, myofibroblast activation, and excessive extracellular matrix deposition [1, 2]. Unilateral ureteral obstruction (UUO) is widely used to mimic obstructive nephropathy and to investigate the early events of tubular epithelial dedifferentiation and interstitial fibrosis [2, 3]. Increasing evidence indicates that tubular epithelial cells actively contribute to fibrotic progression through loss of epithelial polarity, acquisition of mesenchymal features, and secretion of profibrotic mediators [4].

Mesenchymal stem cell–derived exosomes (MSC‐Exos) have emerged as a promising cell‐free therapeutic strategy for tissue repair [5, 6, 7]. Human umbilical cord MSC‐Exos (HucMSC‐Exos) have exhibited potent anti‐inflammatory, anti‐apoptotic, and anti‐fibrotic effects in various organ injury models, including the kidney [8, 9, 10], partly by modulating oxidative stress, mitochondrial function, and immune responses [11, 12, 13]. However, the molecular mechanisms by which exosomes maintain tubular epithelial identity and polarity remain incompletely understood.

Mafb, a member of the large Maf family of transcription factors, has been recognized as a key regulator of cellular differentiation and tissue homeostasis [14, 15, 16]. During kidney development, Mafb is highly expressed in renal epithelial cells and plays an essential role in nephron maturation and maintenance of epithelial polarity [17]. *Mafb* deficiency impairs tubular differentiation and disrupts epithelial architecture [17, 18]. However, its role in kidney injury and fibrosis, particularly in UUO‐induced epithelial dedifferentiation, remains unknown.

In this study, we investigated the role of Mafb in UUO‐induced renal injury and examined whether HucMSC‐Exos exert protective effects through Mafb regulation. We aimed to determine whether Mafb participates in tubular epithelial dedifferentiation and contributes to renal fibrosis, as well as to explore the potential mechanisms underlying HucMSC‐Exo‐mediated renoprotection.

## 2 Materials and Methods

### 2.1 Experimental Animals

*Mafb* knockout (*Mafb* ^−/−^) mice were generated using the CRISPR/Cas9 system in collaboration with Cyagen Biosciences Inc. (Suzhou, China). Wild‐type C57BL/6J mice were purchased from the Laboratory Animal Center of Chongqing Medical University. Genotyping was performed by polymerase chain reaction (PCR) and confirmed by DNA sequencing. All mice were housed under specific‐pathogen‐free (SPF) conditions with free access to food and water at the Laboratory Center of Children's Hospital of Chongqing Medical University. To investigate the role of *Mafb* in the kidney, homozygous *Mafb* ^−/−^ embryos at embryonic day 18.5 (E18.5) and gestational age‐matched wild‐type littermates were used. All animal experiments were approved by the Ethics Committee of Children's Hospital of Chongqing Medical University and conducted in accordance with institutional guidelines (Approval No. CHCMU—IACUC20250429004).

### 2.2 Isolation and Characterization of HucMSC‐Derived Exosomes

Human umbilical cord mesenchymal stem cells (HucMSCs) were cultured in an exosome‐depleted medium. When cells reached approximately 80% confluence, the conditioned medium was collected and subjected to sequential centrifugation at 300 × g for 10 min, 2000 × g for 20 min, and 10,000 × g for 30 min to remove cells and debris. The supernatant was then ultracentrifuged at 100,000 × g for 70 min at 4°C. The exosome pellet was washed once with Phosphate‐buffered saline (PBS; Gibco, Thermo Fisher Scientific, Waltham, MA, USA), ultracentrifuged again at 100,000 × g for 70 min, and finally resuspended in sterile PBS. Exosome morphology was examined by transmission electron microscopy (TEM). Expression of exosomal markers CD63, Alix, and TSG101 and the negative marker calnexin was analyzed by western blotting. Nanoparticle flow cytometry was used to further confirm exosomal surface marker expression. The protein concentration of exosome preparations was determined using a bicinchoninic acid (BCA) protein assay kit (Beyotime Biotechnology, Shanghai, China).

### 2.3 UUO Model and Exosome Administration

Male C57BL/6J mice (8–10 weeks old) were randomly divided into three groups: sham, UUO, and UUO + Exo groups (*n* = 4 per group). Mice in the sham group underwent sham surgery, whereas mice in the UUO and UUO + Exo groups underwent unilateral ureteral obstruction (UUO) surgery. Under general anesthesia, the left ureter was exposed and ligated with 4‐0 silk sutures. Sham‐operated mice underwent the same surgical procedure without ureter ligation. For exosome treatment, mice in the UUO + Exo group received a single intravenous injection of HucMSC‐Exos (100 μg in 100 μL PBS) via the tail vein on the first day after UUO surgery. Mice in the UUO group received an equal volume of PBS. Kidneys were harvested at day 3 post‐surgery (UUO3) for subsequent analyses.

### 2.4 Histology, Immunohistochemistry, and Immunofluorescence

Kidney tissues were fixed in 4% paraformaldehyde, embedded in paraffin, and sectioned at 4 μm thickness. Sections were subjected to hematoxylin–eosin (H&E) and periodic acid–Schiff (PAS) staining according to standard protocols. For immunohistochemistry (IHC) and immunofluorescence (IF) staining, paraffin sections were deparaffinized, rehydrated, and subjected to antigen retrieval. For cell IF staining, cultured Human renal proximal tubular epithelial (HK‐2) cells were fixed with 4% paraformaldehyde for 20 minutes and permeabilized with 0.2% Triton X‐100 for 10 minutes. Samples were blocked with 0.5% bovine serum albumin for 1 hour at room temperature and incubated overnight at 4°C with primary antibodies. After washing, sections or cells were incubated with cyanine 3 (Cy3)‐conjugated goat anti‐rabbit or goat anti‐mouse IgG secondary antibodies (Proteintech, Wuhan, Hubei, China) at a dilution of 1:200 for 1 hour at room temperature. Nuclei were counterstained with Hoechst 33342 (Thermo Fisher, 1:1000). Images were captured using a confocal fluorescence microscope (Nikon).

### 2.5 RNA Isolation and Quantitative Real‐Time PCR (RT‐qPCR)

Total RNA from kidney tissues and cultured cells was extracted using the Simply P Total RNA Extraction Kit (BioFlux). Complementary DNA (cDNA) was synthesized using RT Master Mix (MCE, USA). Quantitative PCR was performed using SYBR GREEN qPCR Master Mix (MCE) on a CFX96 real‐time PCR detection system (Bio‐Rad). The amplification conditions were 95°C for 5 minutes, followed by 40 cycles of 95°C for 15 seconds and 60°C for 1 minute. Relative gene expression was calculated using the 2^−ΔΔCt^ method with glyceraldehyde‐3‐phosphate dehydrogenase (GAPDH) as the internal control. All reactions were performed in triplicate. Primer sequences are listed in Supporting Information S1: Table S1.

### 2.6 Cell Culture, Small Interfering RNA (siRNA) Knockdown, and Exosome Treatment

HK‐2 cells were cultured in Dulbecco’s Modified Eagle Medium/Nutrient Mixture F‐12 (DMEM/F12) supplemented with 10% fetal bovine serum, penicillin (100 U/mL), and streptomycin (100 μg/mL). Cells were maintained at 37°C in a humidified incubator with 5% CO_2_. For *Mafb* knockdown experiments, cells at approximately 50%–60% confluence were transfected with *Mafb*‐specific siRNA or negative control siRNA (50 nmol/L) using Lipofectamine 3000 (Thermo Fisher Scientific, Waltham, MA, USA) according to the manufacturer's protocol. The sequences of *Mafb* siRNA were as follows: sense strand 5′‐CCAUCACCAUCAUCACCAA‐3′ and antisense strand 5′‐UUGGUGAUGAUGGUGAUGG‐3′. After 48 hours of transfection, cells were stimulated with TGF‐β1 (5 ng/mL) for 24–48 hours to induce tubular epithelial dedifferentiation. Where indicated, HucMSC‐derived exosomes (50 μg/mL) were added 12 hours prior to TGF‐β1 stimulation.

### 2.7 Western Blot Analysis

Total proteins from kidney tissues or cultured cells were extracted using radioimmunoprecipitation assay (RIPA) lysis buffer (MCE, USA) containing protease inhibitors. Protein concentrations were determined using a BCA assay (Beyotime, Shanghai, China). Equal amounts of protein (30–50 μg) were separated by SDS‐PAGE and transferred onto polyvinylidene fluoride (PVDF) membranes (Millipore, Billerica, MA, USA). After blocking, membranes were incubated overnight at 4°C with primary antibodies. After incubation with horseradish peroxidase (HRP)‐conjugated secondary antibodies, protein bands were visualized using an enhanced chemiluminescence (ECL) kit (MCE) and imaged with a ChemiDoc Touch Imaging System (Bio‐Rad). Band intensities were quantified using ImageJ software. Detailed information on all primary antibodies used in this study is provided in Supporting Information S1: Table S2.

### 2.8 Statistical Analysis

All experiments were independently repeated at least three times. Data are presented as mean ± standard deviation (SD). Statistical analyses were performed using GraphPad Prism 8.0 software. Comparisons between two groups were made using Student's *t*‐test, and comparisons among multiple groups were analyzed by one‐way ANOVA followed by Tukey's post hoc test. A *p* value < 0.05 was considered statistically significant.

## 3 Results

### 3.1 Characterization of HucMSC‐Derived Exosomes

To verify the successful isolation of human umbilical cord mesenchymal stem cell–derived exosomes (HucMSC‐Exos), a series of morphological and molecular characterizations were performed. Transmission electron microscopy (TEM) revealed that the isolated vesicles exhibited the typical cup‐shaped morphology with diameters ranging from approximately 50 to 150 nm (Figure 1A). Western blot analysis confirmed the presence of canonical exosomal markers CD63, Alix, and TSG101, whereas the endoplasmic reticulum protein calnexin was absent, indicating high purity of the isolated exosomes (Figure 1B). In addition, nanoparticle flow cytometry further validated the expression of exosomal surface markers, supporting the identity of the isolated particles as exosomes (Figure 1C). Collectively, these data demonstrate that the isolated extracellular vesicles meet the established criteria for exosomes and are suitable for subsequent functional experiments.

**FIGURE 1:**
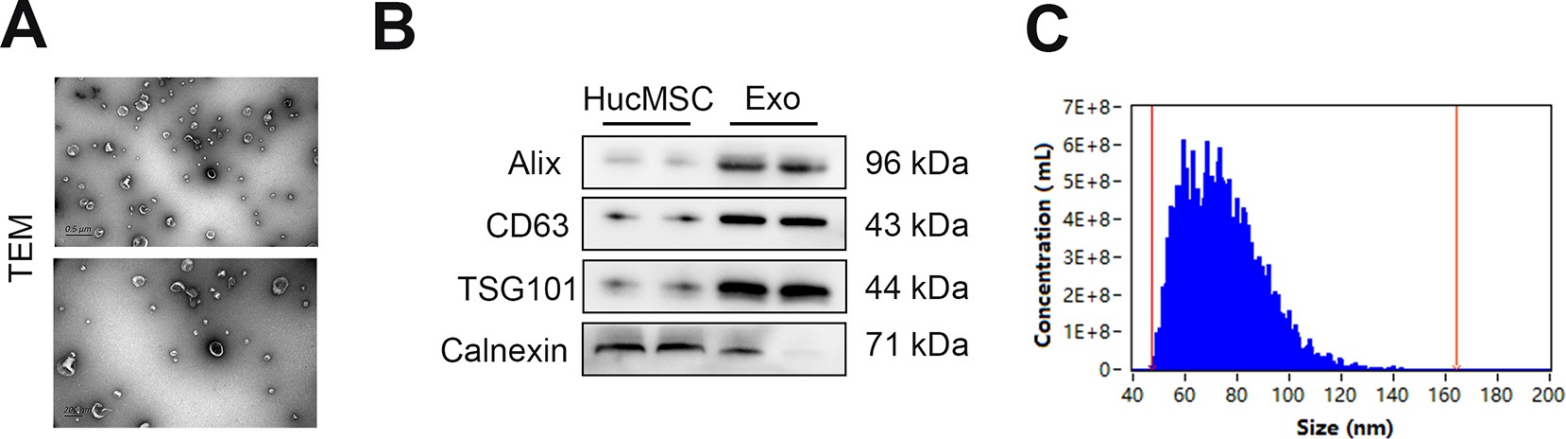
Characterization of HucMSC‐derived exosomes. (A) Representative transmission electron microscopy (TEM) image showing the typical cup‐shaped morphology of isolated HucMSC‐Exos. (B) Representative western blot analysis of exosomal markers CD63, Alix, and TSG101 in HucMSC‐Exos. Calnexin was used as a negative control to confirm the absence of cellular contamination. Protein molecular weights are indicated in kilodaltons (kDa). (C) Flow cytometry analysis confirming the expression of exosomal surface markers. HucMSC, human umbilical cord mesenchymal stem cell.

### 3.2 HucMSC‐Exos Alleviate UUO‐Induced Tubular Epithelial Injury and Dedifferentiation

To evaluate the therapeutic potential of HucMSC‐Exos in renal injury, a UUO mouse model was established, and HucMSC‐Exos or PBS were intravenously administered. Histological analysis at day 3 after UUO revealed marked renal tubular injury, characterized by tubular atrophy, luminal dilation, and basement membrane thickening, as demonstrated by H&E and PAS staining. These pathological alterations were substantially ameliorated in mice treated with HucMSC‐Exos (Figure 2A).

**FIGURE 2:**
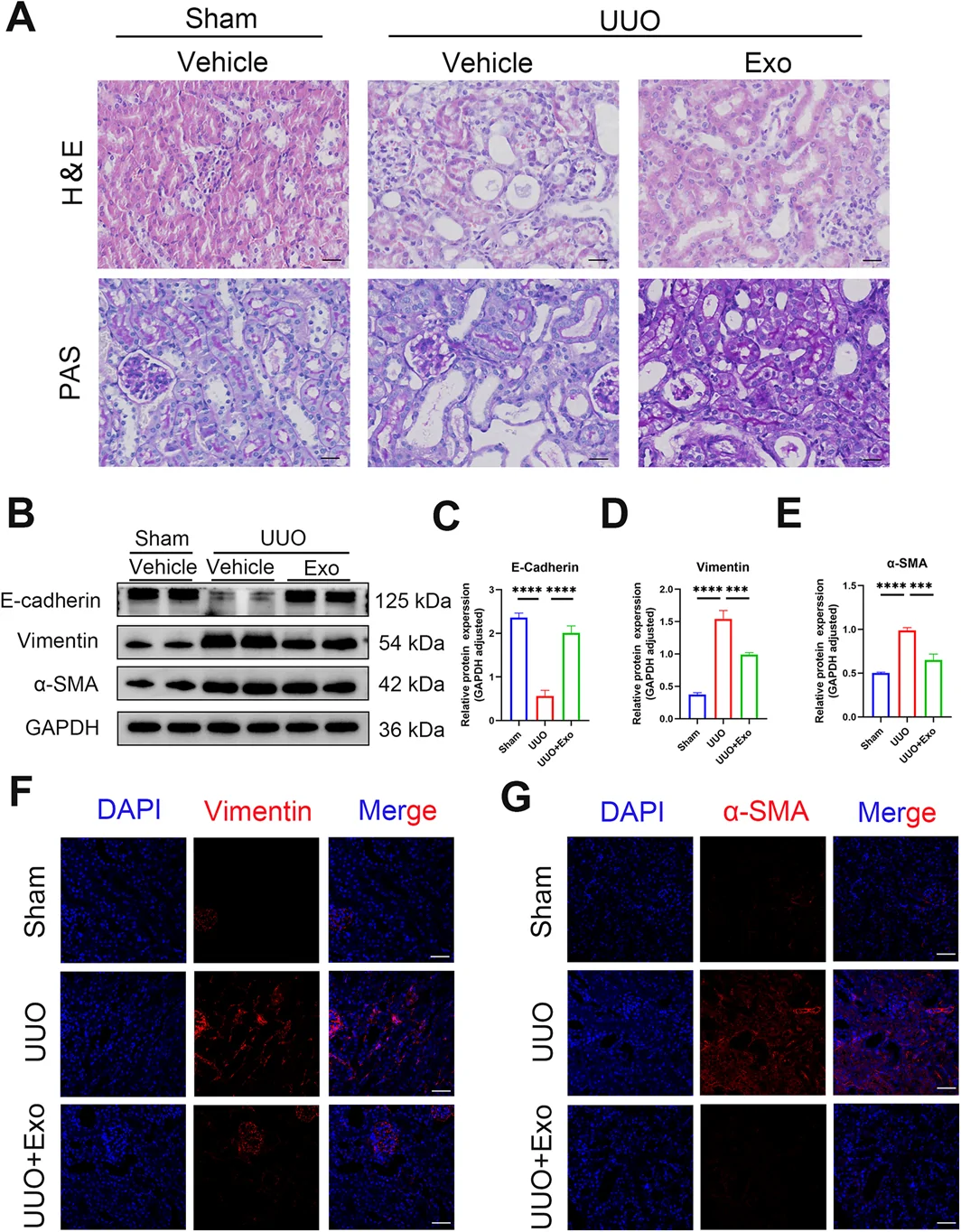
Human umbilical cord mesenchymal stem cell–derived exosomes (HucMSC‐Exos) alleviate unilateral ureteral obstruction (UUO)‐induced tubular epithelial injury and dedifferentiation. (A) Representative hematoxylin and eosin (H&E) and periodic acid‐Schiff (PAS) staining of kidney sections from Sham, UUO, and UUO + Exo groups at day 3 after UUO/sham surgery, showing that HucMSC‐Exo treatment alleviated tubular atrophy and structural disruption. Scale bar = 25 μm. (B) Representative western blot analysis of epithelial marker E‐cadherin and mesenchymal markers vimentin and α‐SMA in kidney tissues. (C–E) Quantitative analysis of protein levels shown in (B). (F) Immunofluorescence staining of vimentin in kidney sections from the indicated groups. (G) Immunofluorescence staining of α‐SMA in kidney sections from the indicated groups. Scale bar = 25 μm. Data are presented as mean ± standard deviation (*n* = 4 mice per group). Statistical analysis was performed using one‐way ANOVA followed by Tukey's post hoc test. *p < 0.05, **p < 0.01, ***p < 0.001, ****p < 0.0001.

Consistent with the histological findings, western blot analysis showed a significant upregulation of mesenchymal markers vimentin and α‐smooth muscle actin (α‐SMA), accompanied by the downregulation of the epithelial marker E‐cadherin in UUO kidneys. Notably, administration of HucMSC‐Exos partially reversed these changes, indicating restoration of epithelial phenotype (Figure 2B–E).

Immunofluorescence staining further confirmed a pronounced expansion of vimentin‐ and α‐SMA–positive areas in UUO kidneys, which was markedly reduced following HucMSC‐Exo treatment (Figure 2F,G). In addition, kidney wet weight was significantly increased after UUO surgery, whereas exosome administration attenuated this increase (Supporting Information S1: Table S3). Moreover, immunofluorescence staining for E‐cadherin and quantitative PCR (qPCR) analyses of E‐cadherin and vimentin showed consistent trends (Supporting Information S1: Figure S1).

Taken together, these results demonstrate that UUO induces early tubular epithelial dedifferentiation and structural disruption, whereas HucMSC‐Exos effectively preserve epithelial integrity and mitigate renal injury.

### 3.3 HucMSC‐Exos Restore Mafb Expression Downregulated in UUO Kidneys

To investigate the potential involvement of Mafb in UUO‐induced renal injury and the effects of HucMSC‐Exos, we first examined Mafb expression in kidney tissues in the Sham, UUO, and UUO + Exo groups. qPCR analysis revealed that *Mafb* mRNA levels were significantly decreased in UUO kidneys compared with the Sham group, whereas treatment with HucMSC‐Exos markedly restored *Mafb* levels (Figure 3A). Consistently, western blot analysis demonstrated a substantial reduction of Mafb protein in UUO kidneys, which was partially reversed following exosome administration (Figure 3B,C).

**FIGURE 3:**
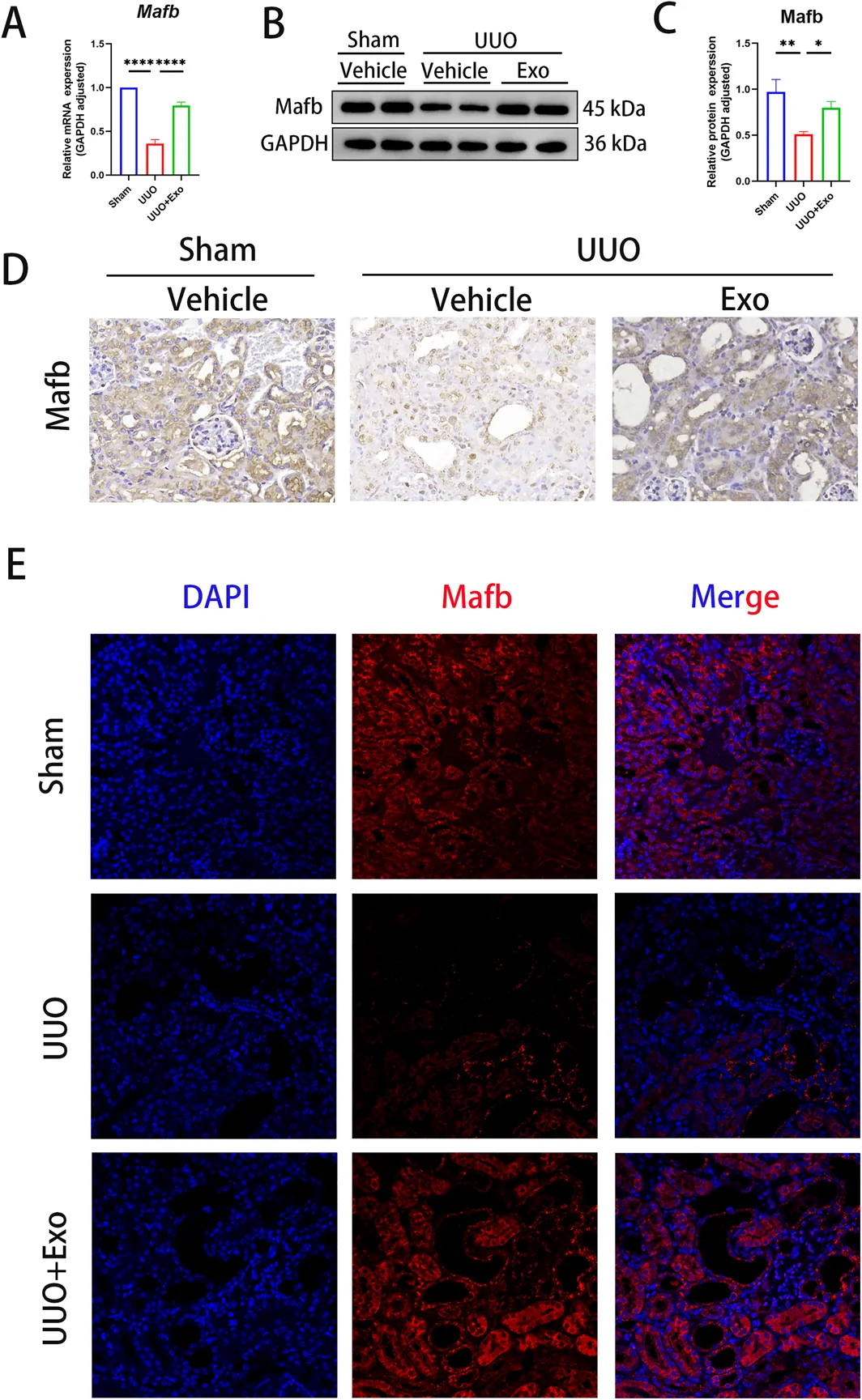
Human umbilical cord mesenchymal stem cell–derived exosomes (HucMSC‐Exos) restore Mafb expression downregulated in unilateral ureteral obstruction (UUO) kidneys. (A) Quantitative PCR analysis of *Mafb* mRNA levels in kidney tissues from Sham, UUO, and UUO + Exo groups. (B) Representative western blot analysis of Mafb protein expression in the indicated groups. (C) Quantification of Mafb protein levels shown in (B). (D) Representative immunohistochemical staining of Mafb in kidney sections. Scale bar = 25 μm. (E) Immunofluorescence staining of Mafb in renal tubules. Scale bar = 25 μm. Data are presented as mean ± standard deviation (*n* = 4 mice per group). Statistical analysis was performed using one‐way ANOVA followed by Tukey's post hoc test. *p < 0.05, **p < 0.01, ***p < 0.001, ****p < 0.0001.

Immunohistochemical staining further confirmed that Mafb expression was predominantly localized in tubular epithelial cells in Sham kidneys. After UUO, Mafb‐positive signals were markedly diminished and became scattered in degenerative tubules, whereas HucMSC‐Exo treatment restored Mafb expression within the tubular compartment (Figure 3D). Immunofluorescence staining yielded similar results, demonstrating the recovery of Mafb expression following exosome therapy (Figure 3E).

These findings suggest that Mafb downregulation is closely associated with UUO‐induced tubular dedifferentiation and that HucMSC‐Exos can effectively rescue Mafb expression in injured kidneys.

### 3.4 *Mafb* Knockdown Aggravates TGF‐β–Induced Epithelial Dedifferentiation in HK‐2 Cells

To further determine the functional role of Mafb in tubular epithelial plasticity, Mafb expression was silenced in HK‐2 cells using specific siRNA. qPCR analysis confirmed the efficient knockdown of *Mafb* compared with negative control small interfering RNA (si‐NC) (Figure 4A).

**FIGURE 4:**
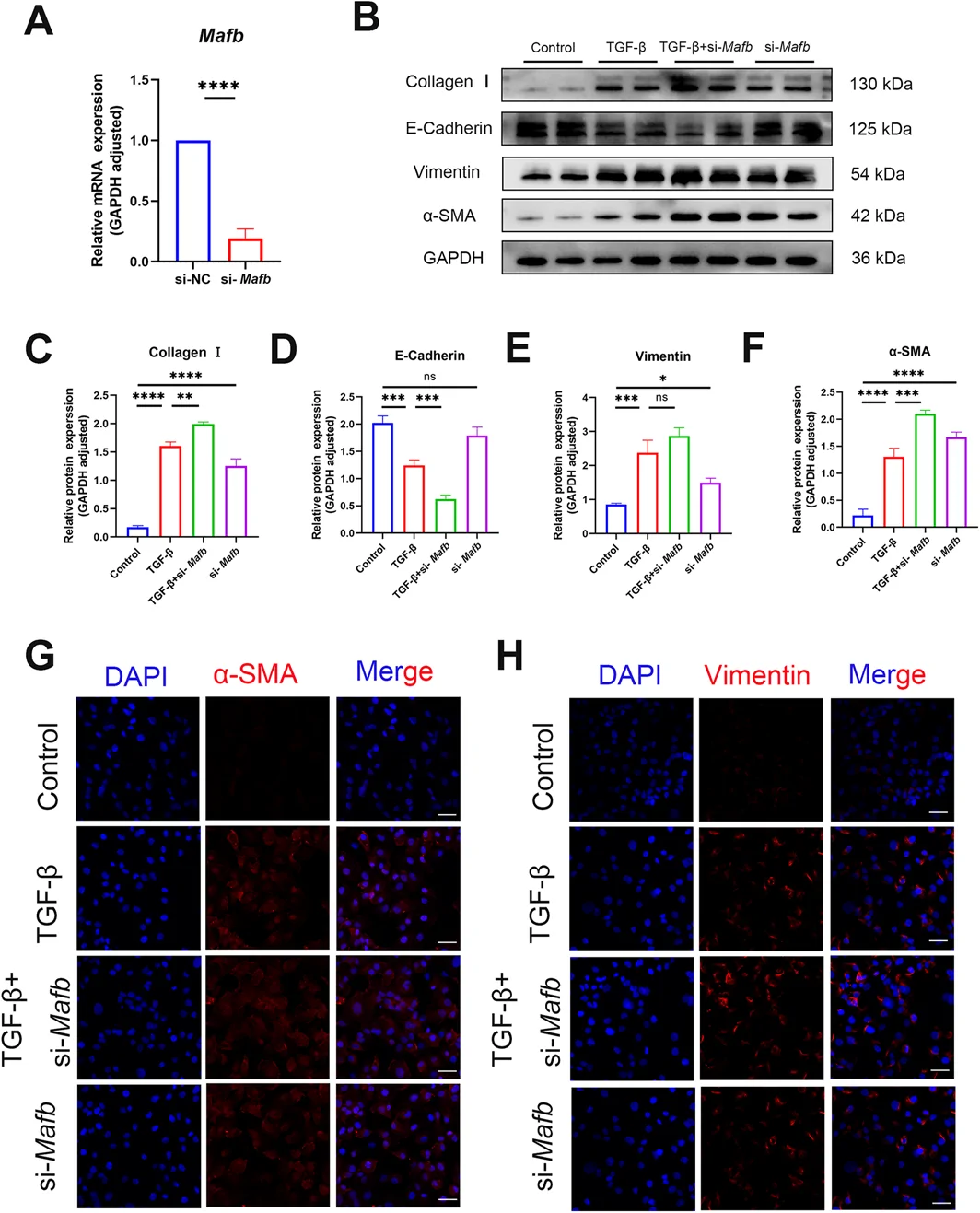
*Mafb* knockdown enhances TGF‐β–induced dedifferentiation in HK‐2 cells. (A) Quantitative PCR analysis confirming *Mafb* knockdown efficiency in HK‐2 cells transfected with *Mafb*‐specific small interfering RNA (si‐*Mafb*). (B) Representative western blot analysis of Collagen I, E‐cadherin, α‐SMA, and vimentin in control, TGF‐β–treated, TGF‐β + si‐*Mafb*, and si‐*Mafb* groups. (C–F) Quantification of protein levels shown in (B). (G) Immunofluorescence staining of α‐SMA in the indicated groups. Scale bar = 25 μm. (H) Immunofluorescence staining of vimentin in the indicated groups. Scale bar = 25 μm. Data are presented as mean ± standard deviation from four independent experiments (*n* = 4). Statistical analysis was performed using one‐way ANOVA followed by Tukey's post hoc test. *p < 0.05, **p < 0.01, ***p < 0.001, ****p < 0.0001.

Upon stimulation with TGF‐β1, HK‐2 cells exhibited typical features of epithelial dedifferentiation, including downregulation of E‐cadherin and upregulation of Collagen I, α‐SMA, and vimentin. Importantly, these changes were significantly exacerbated in *Mafb*‐silenced cells, indicating that the loss of *Mafb* enhances TGF‐β–induced mesenchymal transition (Figure 4B–F).

Immunofluorescence staining further demonstrated increased α‐SMA and vimentin expression in TGF‐β–treated cells, and this effect was markedly intensified following *Mafb* knockdown (Figure 4G,H). Collectively, these results demonstrate that *Mafb* acts as a key negative regulator of TGF‐β–mediated epithelial dedifferentiation in renal tubular cells.

### 3.5 *Mafb* Deficiency Leads to Renal Developmental Defects and Loss of Epithelial Polarity

To explore the in vivo role of *Mafb* in kidney development, kidneys from wild‐type and *Mafb* knockout embryos at E18.5 were analyzed. H&E staining revealed severe developmental abnormalities in *Mafb*‐deficient kidneys, including reduced nephron density, disorganized tubular architecture, and immature glomerular structures (Figure 5A). These morphological defects indicate that *Mafb* is essential for normal renal structural maturation.

To verify the effective deletion of *Mafb*, supplementary validation experiments were performed. Immunohistochemistry, qPCR, and immunofluorescence analyses confirmed markedly decreased *Mafb* mRNA and Mafb protein expression at both transcriptional and protein levels in *Mafb* ^−/−^ kidneys (Supporting Information S1: Figure S2A–C), demonstrating successful gene knockout.

Western blot analysis showed a significant reduction in the epithelial marker E‐cadherin in Mafb‐deficient kidneys compared with wild‐type controls (Figure 5B,C). Consistently, immunofluorescence staining confirmed diminished E‐cadherin expression in tubular epithelium (Figure 5D), indicating impaired epithelial integrity. Furthermore, immunohistochemical staining revealed a markedly reduced expression of the tight junction protein ZO‐1 in Mafb knockout kidneys (Figure 5E). This finding was further supported by additional immunofluorescence analysis presented in Supporting Information S1: Figure S2D, confirming loss of epithelial polarity in the absence of *Mafb*.

Given the pivotal role of Wingless/Integrated (Wnt) signaling pathway in nephrogenesis and epithelial organization, we next examined the expression of Wnt5a and β‐catenin. Western blot and quantitative analyses demonstrated the significant downregulation of both proteins in *Mafb*‐deficient kidneys (Figure 5F–H). Immunofluorescence staining further confirmed reduced Wnt5a and β‐catenin signals in the knockout group (Figure 5I).

**FIGURE 5:**
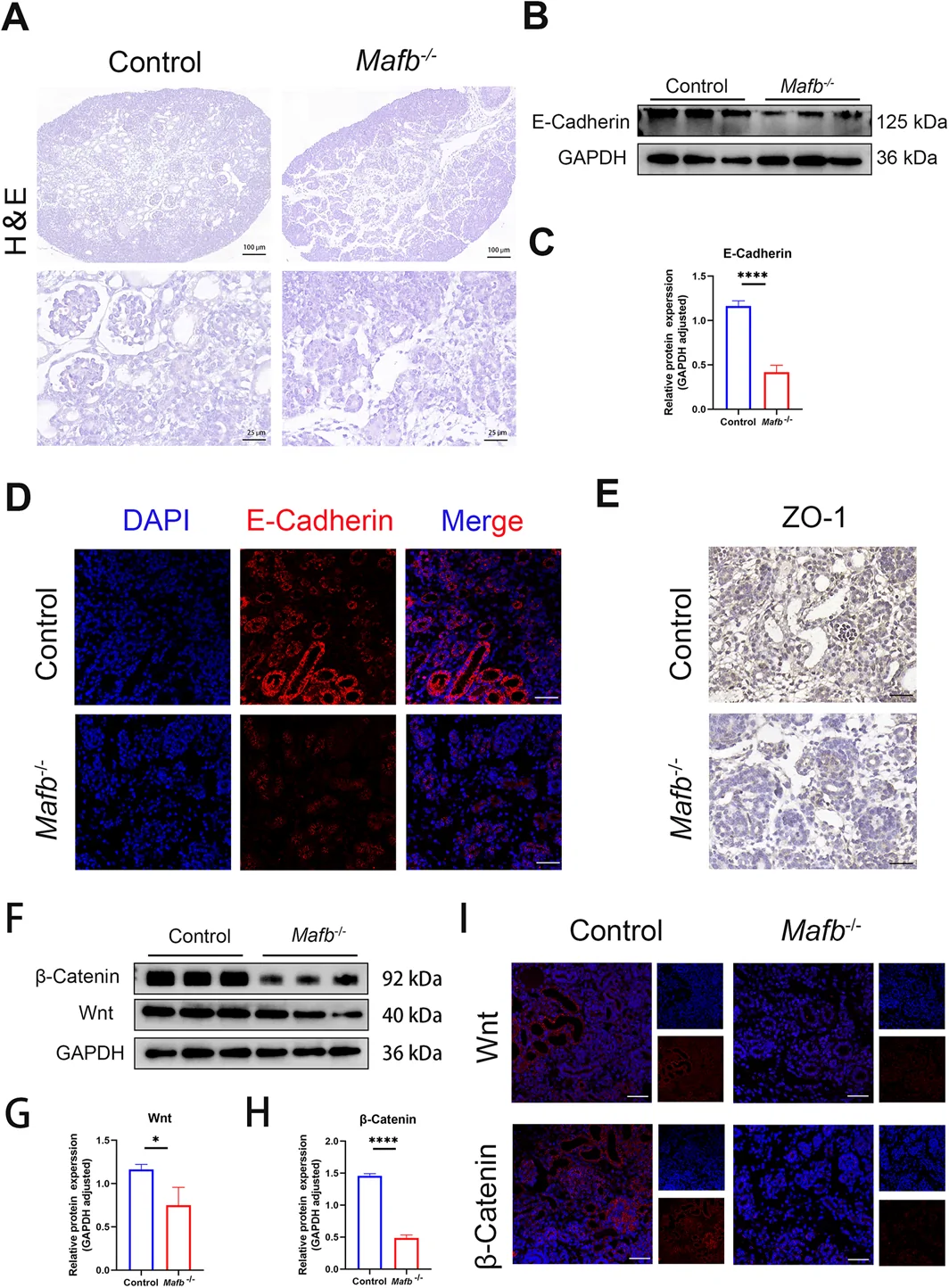
*Mafb* deficiency leads to renal developmental defects and loss of epithelial polarity. (A) Representative hematoxylin and eosin (H&E) staining of kidneys from wild‐type and *Mafb* knockout embryos at embryonic day 18.5, showing developmental defects in *Mafb*‐deficient kidneys. (B) Representative western blot analysis of E‐cadherin expression in control and *Mafb* ^−/−^ kidneys. (C) Quantification of E‐cadherin protein levels shown in (B). (D) Immunofluorescence staining of E‐cadherin in the indicated groups. Scale bar = 25 μm. (E) Immunohistochemical staining of ZO‐1 in the indicated groups. Scale bar = 25 μm. (F) Representative western blot analysis of Wnt5a and β‐catenin in control and *Mafb* ^−/−^ kidneys. (G–H) Quantification of protein levels shown in (F). (I) Immunofluorescence staining of Wnt5a and β‐catenin in the indicated groups. Scale bar = 25 μm. Data are presented as mean ± standard deviation (*n* = 4 mice per group). Statistical analysis was performed using Student's *t*‐test. *p < 0.05, **p < 0.01, ***p < 0.001, ****p < 0.0001.

Taken together, these results demonstrate that Mafb is indispensable for maintaining epithelial polarity and normal renal development, potentially through regulation of the Wnt/β‐catenin signaling pathway.

### 3.6 HucMSC‐Exos Restore Epithelial Polarity in *Mafb*‐Deficient Tubular Cells

To determine whether HucMSC‐Exos can compensate for *Mafb* deficiency, HK‐2 cells with *Mafb* knockdown were treated with TGF‐β1 in the presence or absence of exosomes. Western blot analysis showed that *Mafb* silencing markedly aggravated TGF‐β–induced loss of epithelial markers E‐cadherin and ZO‐1 and increased vimentin expression. Notably, HucMSC‐Exo treatment significantly reversed these alterations, restoring epithelial marker expression and suppressing mesenchymal transition (Figure 6A–D).

Immunofluorescence staining further confirmed that exosome treatment rescued the disrupted epithelial phenotype in *Mafb*‐silenced cells, as evidenced by decreased vimentin and recovery of ZO‐1 localization (Figure 6E,F). qPCR analysis of vimentin expression showed consistent trends with protein results (Figure 6G).

**FIGURE 6:**
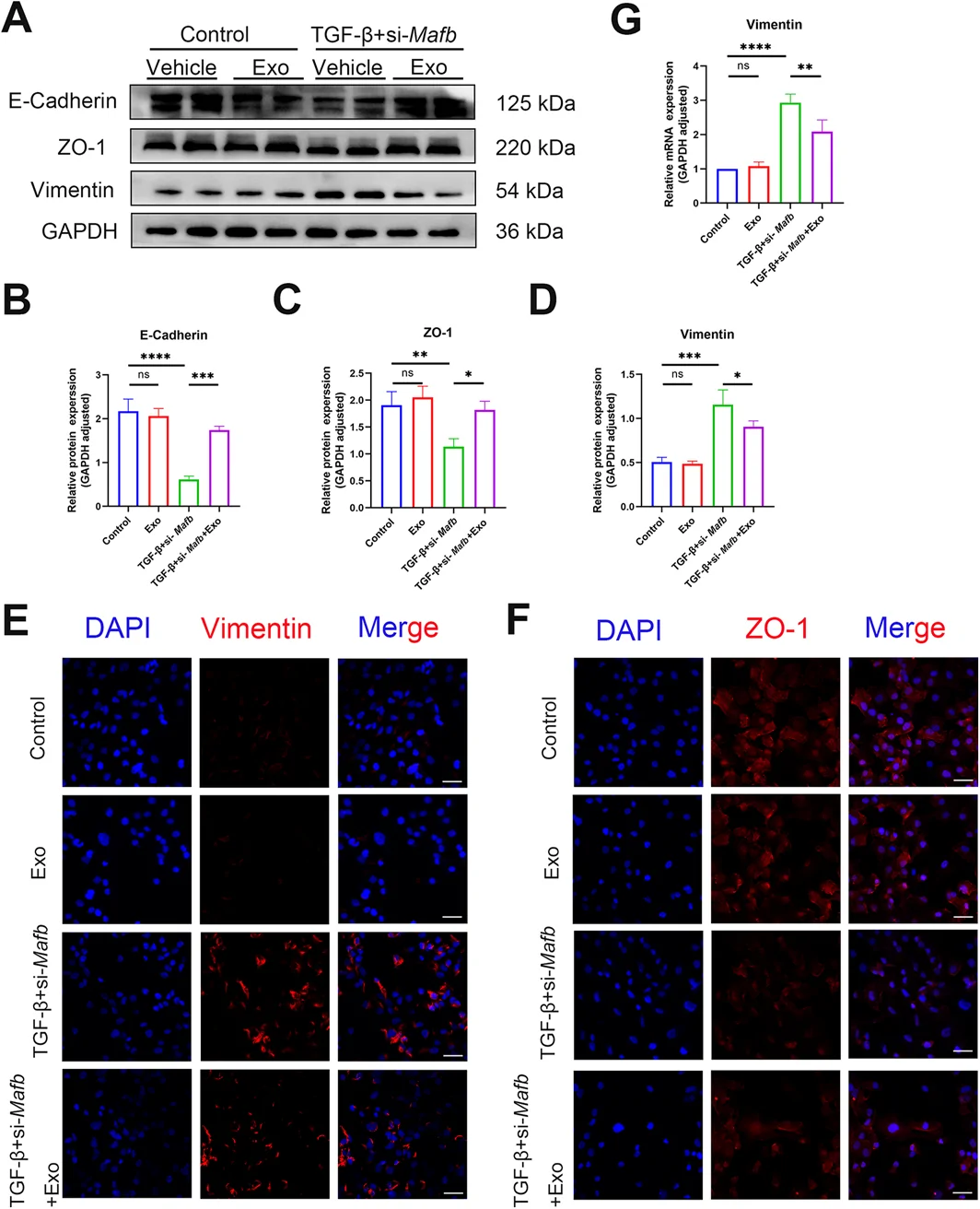
Human umbilical cord mesenchymal stem cell–derived exosomes (HucMSC‐Exos) restore epithelial polarity in Mafb‐deficient tubular cells. (A) Representative western blot analysis of E‐cadherin, ZO‐1, and vimentin in control, Exo, TGF‐β + si‐*Mafb*, and TGF‐β + si‐*Mafb* + Exo groups. (B–D) Quantitative analysis of protein expression shown in (A). (E) Immunofluorescence staining of vimentin in the indicated groups. Scale bar = 25 μm. (F) Immunofluorescence staining of ZO‐1 in the indicated groups. Scale bar = 25 μm. (G) Quantitative PCR analysis of vimentin expression in the indicated groups. Data are derived from four independent experiments (*n* = 4). Data are presented as mean ± standard deviation. Statistical analysis was performed using one‐way ANOVA followed by Tukey's post hoc test. *p < 0.05, **p < 0.01, ***p < 0.001, ****p < 0.0001.

These findings indicate that HucMSC‐Exos can effectively counteract *Mafb* deficiency–induced epithelial injury and dedifferentiation, highlighting a Mafb‐independent protective mechanism of exosome therapy.

## 4 Discussion

In the present study, we identified Mafb as a previously unrecognized transcriptional regulator involved in UUO‐induced tubular epithelial injury and demonstrated that HucMSC‐derived exosomes exert renoprotective effects partially through the restoration of Mafb expression. Our findings provide new mechanistic insights into the maintenance of epithelial polarity during renal fibrosis and highlight Mafb as a potential therapeutic target.

A major finding of this study is the identification of Mafb as a critical factor that is markedly downregulated during UUO‐induced renal injury [19, 20]. Although Mafb has been extensively studied in the context of kidney development, its role in kidney disease has remained largely unexplored [14]. Here, we demonstrate that *Mafb* mRNA levels and MafB protein expression are significantly reduced in UUO kidneys and that this reduction predominantly occurs in tubular epithelial cells. Importantly, the loss of *Mafb* correlates with epithelial dedifferentiation and structural disruption, suggesting that Mafb is closely associated with the maintenance of tubular epithelial identity.

Functional studies in HK‐2 cells further support this notion. Silencing of *Mafb* significantly aggravated TGF‐β–induced loss of E‐cadherin and upregulation of mesenchymal markers, indicating that Mafb acts as an intrinsic suppressor of epithelial dedifferentiation. These results establish Mafb as a novel regulator of tubular epithelial plasticity in the context of renal fibrosis.

Another important observation of this study is the indispensable role of Mafb in renal epithelial development and polarity maintenance. Using *Mafb* knockout embryos, we found that *Mafb* deficiency leads to profound developmental abnormalities, including impaired nephron formation, disorganized tubular structures, and loss of epithelial polarity markers such as E‐cadherin and ZO‐1. These findings are consistent with previous reports that Mafb regulates epithelial differentiation programs.

Furthermore, we observed the significant downregulation of Wnt and β‐catenin signaling in *Mafb*‐deficient kidneys, suggesting that Mafb may maintain epithelial integrity at least in part through the modulation of Wnt signaling pathways. Given the critical role of Wnt/β‐catenin in nephrogenesis and epithelial homeostasis, our data imply that Mafb functions as an upstream transcriptional regulator that safeguards the epithelial architecture. This developmental evidence strengthens the concept that Mafb is fundamentally required for epithelial polarity, and its loss in adult kidneys may predispose to dedifferentiation and fibrotic progression.

A key translational implication of our study is that HucMSC‐Exos effectively restore Mafb expression and thereby ameliorate tubular epithelial injury. In mice with UUO, exosome administration significantly rescued Mafb levels and reversed epithelial dedifferentiation, as evidenced by the recovery of E‐cadherin and reduction of vimentin and α‐SMA. These results suggest that Mafb restoration represents an important mechanism underlying exosome‐mediated renal protection.

Notably, exosome treatment also attenuated epithelial injury in *Mafb*‐silenced HK‐2 cells, indicating that the protective effects of HucMSC‐Exos are not solely dependent on Mafb. This observation implies the existence of Mafb‐independent pathways, potentially involving other exosomal cargos such as microRNAs, mitochondrial regulatory factors, or anti‐inflammatory mediators. Therefore, Mafb likely acts as a major but not exclusive downstream effector of HucMSC‐Exos. Future studies are needed to identify the specific exosomal components responsible for Mafb regulation and to delineate additional protective mechanisms.

Collectively, our study reveals a previously unappreciated role of Mafb in renal fibrosis and identifies Mafb as a mechanistic link between exosome therapy and preservation of epithelial integrity. Targeting Mafb or its downstream pathways may represent a novel strategy for preventing tubular epithelial dedifferentiation and slowing fibrotic progression [15]. Given the growing interest in MSC‐derived exosomes as therapeutic agents, understanding how exosomes regulate key transcriptional programs such as Mafb will facilitate the development of more precise and effective interventions for CKD.

Several limitations of this study should be acknowledged. First, although we demonstrated that exosomes restore Mafb expression, the specific exosomal cargos responsible for this regulation remain to be identified. Second, whether *Mafb* directly regulates epithelial polarity genes or acts indirectly through signaling pathways such as Wnt/β‐catenin requires further mechanistic investigation. Finally, validation in additional models of chronic kidney disease will be necessary to determine the broader relevance of the Mafb–exosome axis.

An important methodological consideration in this study is the choice of experimental models to investigate the function of Mafb in renal fibrosis. Although genetic knockout models are widely used to define gene function in vivo, it has been reported that the global deletion of Mafb results in severe developmental defects and perinatal lethality, with most *Mafb*‐null mice dying shortly after birth due to multi‐organ abnormalities, including profound renal dysgenesis [14, 21]. Consistently, our analysis of *Mafb* ^−/−^ embryos at E18.5 revealed striking defects in nephron formation and epithelial polarity, further confirming the indispensable role of Mafb during kidney development.

Future studies focusing on the upstream regulators of Mafb, the transcriptional targets of Mafb in tubular cells, and the precise exosomal components mediating Mafb restoration will provide deeper insights into the therapeutic potential of this pathway.

## 5 Conclusion

In summary, we demonstrate for the first time that Mafb is a key transcription factor that safeguards tubular epithelial polarity and is critically involved in UUO‐induced renal injury. HucMSC‐derived exosomes alleviate epithelial dedifferentiation and renal fibrosis partially by restoring Mafb expression, whereas additional Mafb‐independent mechanisms also contribute to their protective effects. These findings establish a novel exosome–Mafb regulatory axis and provide a promising therapeutic framework for the treatment of renal fibrosis.

## Author Contributions

All authors contributed to the conception and design of the study. Zhuocheng Shi performed the majority of the experiments and drafted the manuscript. Chenxi Jia participated in data analysis and interpretation. Meiling Chen assisted with the animal experiments. Deying Zhang conceived the study, supervised the project, and critically revised the manuscript. All authors read and approved the final version of the manuscript.

## Funding

This work was supported by the Chongqing Key Project of Technology Innovation and Application (Grant No. 2024TIAD‐KPX0035), the Joint Project of Chongqing Health Commission and Science & Technology Bureau (Grant No. 2025ZDXM038), and the Chongqing Medical University Program for Youth Innovation in Future Medicine (Grant No. W0056).

## Ethics Statement

All animal experiments were conducted in accordance with the Basel Declaration and the relevant regulations for the management and ethical treatment of laboratory animals in China. The study protocol was reviewed and approved by the Ethics Committee of Chongqing Medical University (Approval No. CHCMU‐IACUC20250429004).

## Conflicts of Interest

The authors declare no conflicts of interest.

## Supporting information


Supporting Information S1

## Data Availability

The datasets generated and/or analyzed during the current study are available from the corresponding author upon reasonable request.
