# Supplementary material for: HucMSC‐Derived Exosomes Preserve Mafb‐Dependent Tubular Epithelial Identity and Suppress Dedifferentiation in Obstructive Nephropathy
Source: Pediatr Discov. 2026 Jun 30:e70059. Online ahead of print. doi: 10.1002/pdi3.70059 (PMC13398965; doi:10.1002/pdi3.70059)
Supplement: Supplementary file 1 — Supporting Information S1 [file PDI3-9999-0-s001.doc]

**Supplementary Figure S1. HucMSC-Exos alleviate UUO-induced tubular epithelial injury and dedifferentiation**

**
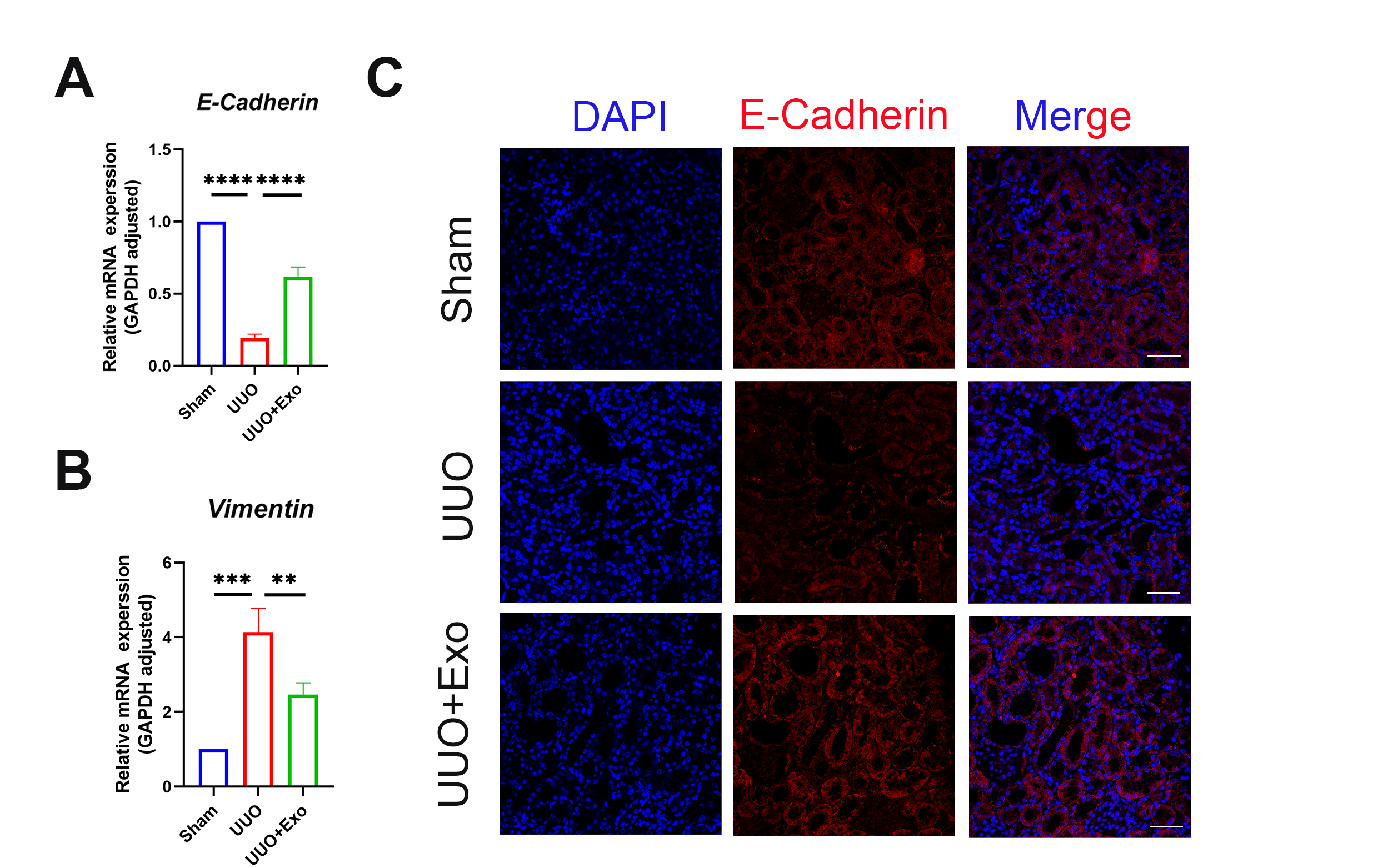
**

**Supplementary Figure S1. HucMSC-Exos alleviate UUO-induced tubular epithelial injury and dedifferentiation.**
(A)Quantitative PCR analysis of *Vimentin* mRNA expression in kidneys from Sham, UUO3, and UUO3+HucMSC-Exo groups. (B) Quantitative PCR analysis of *E-cadherin* mRNA expression in kidneys from Sham, UUO3, and UUO3+HucMSC-Exo groups. (C)Representative immunofluorescence staining of E-cadherin in kidney sections from Sham, UUO3, and UUO3+HucMSC-Exo groups. Scale bar = 25μm. Data are presented as mean ± standard deviation. **p* < 0.05, ***p* < 0.01, ****p* < 0.001, *****p*< 0.0001.

**Supplementary Figure S2. *Mafb* deficiency leads to renal developmental defects and loss of epithelial polarity**

**
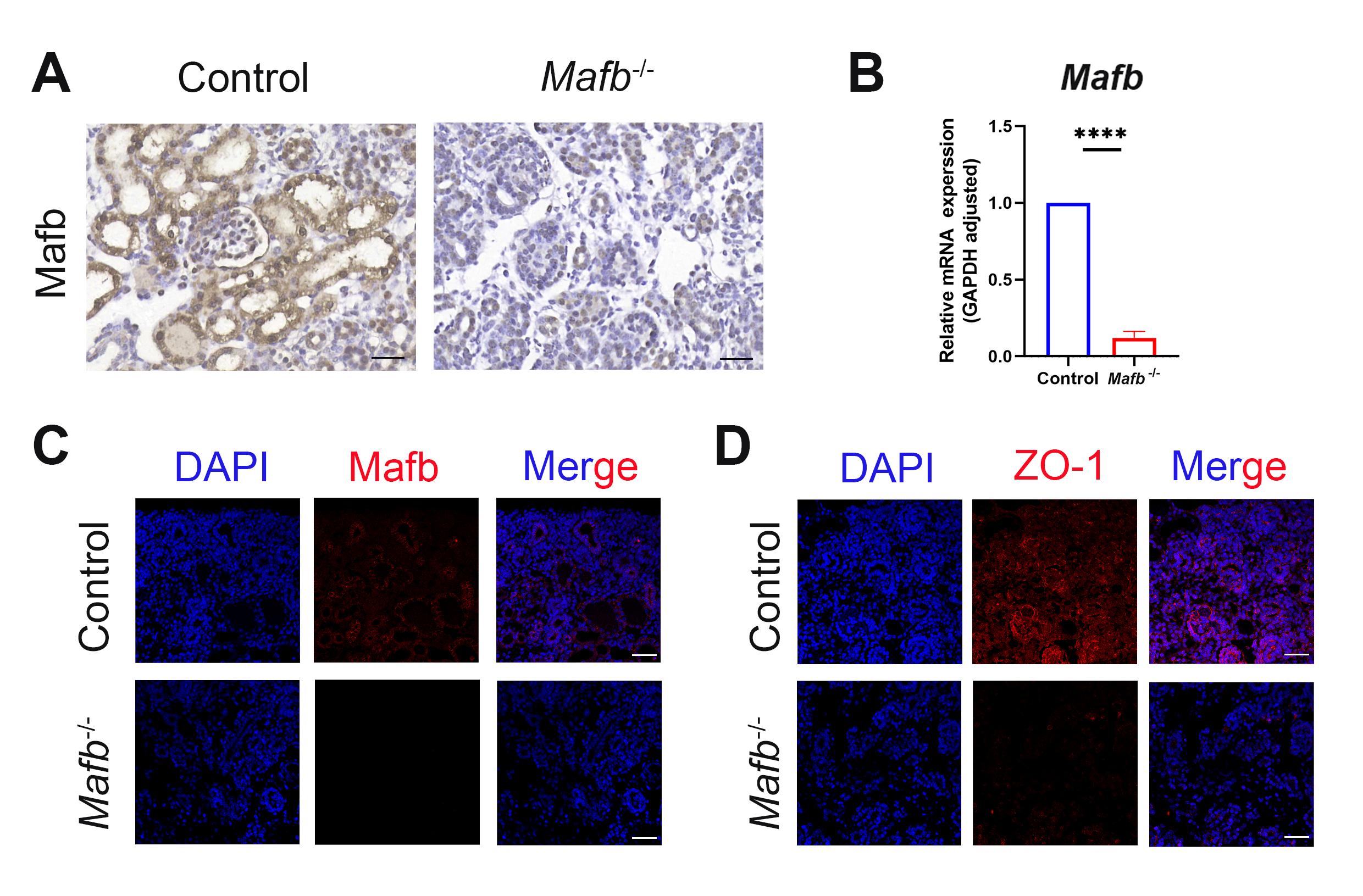
**

**Supplementary Figure S2. *Mafb* deficiency leads to renal developmental defects and loss of epithelial polarity.**
(A) Representative immunohistochemical staining of Mafb in kidney sections from wild-type and *Mafb*⁻/⁻ embryos at embryonic day 18.5 (E18.5), showing markedly reduced Mafb expression in knockout kidneys. Scale bar = 25μm. (B) Quantitative PCR analysis confirming significant downregulation of *Mafb* mRNA in *Mafb*⁻/⁻ kidneys compared with wild-type controls. (C) Immunofluorescence staining of Mafb in E18.5 kidneys, further validating loss of Mafb protein expression in *Mafb*-deficient mice. Scale bar =25μm. (D) Immunofluorescence staining of the tight junction protein ZO-1 demonstrating disrupted epithelial polarity in *Mafb*⁻/⁻ kidneys. Scale bar = 25μm. Data are presented as mean ± standard deviation. **p* < 0.05, ***p* < 0.01, ****p* < 0.001, *****p*< 0.0001.

****Supplementary Table S1.** Real-time qPCR primer sequences for mRNA targets in mouse and human.**

| **Species** | **Target** | **Primer name** | **Sequence (5′ → 3′)** |
| --- | --- | --- | --- |
| **Mouse** | **Mafb** | **Mafb**-F | **GAGCAGCAGAGTGGAGATGA** |
|  |  | **Mafb**-R | **CCTGCTGGTAGGAGGTGTTG** |
| **Mouse** | **E-cadherin** | **E-cadherin**-F | **TGCCTCCTGAAAAGAGAGTGGA** |
|  |  | **E-cadherin**-R | **TGGGGTCTTCTTCTGTTGGC** |
| **Mouse** | **Vimentin** | **Vimentin**-F | **GACGCCATCAACACCGAGTT** |
|  |  | **Vimentin**-R | **CTTGTCGTTGGTTAGCTGGT** |
| **Mouse** | **GAPDH** | **GAPDH**-F | **AGGTCGGTGTGAACGGATTTG** |
|  |  | **GAPDH**-R | **TGTAGACCATGTAGTTGAGGTCA** |
| **Human** | **Mafb** | **Mafb**-F | **CCAGGAGAAGAGGAGGAGGA** |
|  |  | **Mafb**-R | **TGATGGCTGGGATGAGGTAG** |
| **Human** | **GAPDH** | **GAPDH**-F | **GGAGCGAGATCCCTCCAAAAT** |
|  |  | **GAPDH**-R | **GGCTGTTGTCATACTTCTCAT** |

**Supplementary Table S2. List of primary antibodies used in this study.**

| **Antibody** | **Catalog Number** | **Manufacturer** | **Host Species** | **Dilution** | **RRID** |
| --- | --- | --- | --- | --- | --- |
| **E-Cadherin** | **A20798** | **ABclonal** | **Rabbit** | **1:200** | **N/A** |
| **Vimentin** | **R22775** | **Zenbio** | **Rabbit** | **1:500** | **N/A** |
| **α-SMA** | **A24146** | **ABclonal** | **Rabbit** | **1:200** | **N/A** |
| **Collagen Ⅰ** | **A21069** | **ABclonal** | **Rabbit** | **1:200** | **N/A** |
| **CD63** | **A19023** | **ABclonal** | **Rabbit** | **1:1000** | **N/A** |
| **TSG101** | **A1692** | **ABclonal** | **Rabbit** | **1:1000** | **N/A** |
| **Alix** | **A25326** | **ABclonal** | **Rabbit** | **1:1000** | **N/A** |
| **Calnexin** | **A4846** | **ABclonal** | **Rabbit** | **1:1000** | **N/A** |
| **GAPDH** | **A19056** | **ABclonal** | **Rabbit** | **1:1000** | **N/A** |
| **Mafb** | **20189-1-AP** | **Proteintech** | **Rabbit** | **1:200** | **AB_10599005** |
| **Wnt5a** | **P41221** | **Zenbio** | **Rabbit** | **1:200** | **N/A** |
| **β-Catenin** | **P35222** | **Zenbio** | **Rabbit** | **1:200** | **N/A** |
| **ZO-1** | **P39447** | **ABclonal** | **Rabbit** | **1:200** | **N/A** |

**Supplementary Table S3**. Body weight, obstructed kidney wet weight, and kidney index in experimental mice

| Group | Body weight (g, mean ± SD, *n* = 4 ) | Right (obstructed) kidney wet weight (g, mean ± SD) | Kidney index (%, mean ± SD) |
| --- | --- | --- | --- |
| Sham | **22.30 ± 0.65** | **0.118 ± 0.004** | **0.529 ± 0.018** |
| UUO | **19.52 ± 0.74** | **0.196 ± 0.007** | **1.004 ± 0.054** |
| UUO+HucMSC-Exo | **20.18 ± 0.41** | **0.178 ± 0.005** | **0.883 ± 0.031** |

**Supplementary Table S3. Body weight, obstructed kidney wet weight, and kidney index in experimental mice.
Data are presented as mean ± SD. Kidney index was calculated as: (obstructed kidney wet weight / body weight) × 100%. Right kidney wet weight represents the obstructed kidney in UUO groups. Compared with the UUO3 group, HucMSC-Exo treatment partially reduced obstructed kidney enlargement, as reflected by decreased kidney wet weight and kidney index.**
